# Similarities and Distinctions between Cortical Neural Substrates That Underlie Generation of Malevolent Creative Ideas

**DOI:** 10.1523/ENEURO.0127-23.2023

**Published:** 2023-09-18

**Authors:** Xinuo Qiao, Kelong Lu, Qiang Yun, Ning Hao

**Affiliations:** 1 Shanghai Key Laboratory of Mental Health and Psychological Crisis Intervention, School of Psychology and Cognitive Science, East China Normal University, Shanghai 200062, People’s Republic of China; 2 School of Mental Health, Wenzhou Medical University, Wenzhou Zhejiang, 325035, People’s Republic of China

**Keywords:** creativity, fNIRS, functional connectivity, malevolent creativity

## Abstract

Creativity can be driven by negative intentions, and this is called malevolent creativity (MC). It is a type of creativity that serves antisocial purposes and deliberately leads to harmful or immoral results. A possible classification indicates that there are three kinds of MC in daily life: hurting people, lying, and playing tricks. This study aimed to explore similar and distinct neural substrates underlying these different kinds of MC idea generation. The participants were asked to perform different MC tasks, and their neural responses were recorded using a functional near-infrared spectroscopy device. The findings revealed that most regions within the prefrontal and temporal lobes [e.g., the right dorsolateral prefrontal cortex (rDLPFC), and right angular gyrus] were involved in the three MC tasks. However, the right frontopolar cortex (rFPC) was more activated and less coupled with the rDLPFC and right precuneus during the lying task than during the other tasks. Thus, rFPC may play an important role in constructing novel lies. In the lying task, individuals were more selfish and less compassionate. In the playing tricks and hurting people tasks, there was less neural coupling between the rDLPFC and the left inferior frontal gyrus/right inferior parietal lobule than that in the lying task. This may imply that selfish motivation is released when individuals try to ignore victims’ distress or generate aggressive tricks in hurting people or playing tricks tasks. These findings indicate that the three kinds of MC idea generation involve common cortical regions related to creative idea generation and moral judgment, whereas differences in cortical responses exist because of their unique features.

## Significance Statement

Malevolent creativity (MC) is a type of creativity that serves antisocial purposes and deliberately leads to harmful or immoral results. A possible classification indicates that there are three kinds of MC in daily life: hurting people, lying, and playing tricks. The present study, for the first time, explored the similarities and distinctions between cortical neural substrates that underlie generation of malevolent creative ideas. The findings indicate that the three kinds of MC idea generation involve common cortical regions related to creative idea generation and moral judgment, whereas differences in cortical responses exist because of their unique features. This study provided support for the MC three-dimensional framework (i.e., hurting people, lying, and playing tricks), and preliminarily revealed the underlying neural substrates of various MC idea generation, which helps to develop effective techniques or approaches to manage and prevent malevolent creative behaviors.

## Introduction

Creativity is the ability to produce novel and useful work that usually contributes to positive outcomes (Sternberg and Lubart, 1996; Runco and Acar, 2012), but it can also be driven by evil intentions, resulting in negative outcomes. For example, an employee may tell a creative lie to steal credit from their colleagues. This creativity is termed malevolent creativity (MC), serves antisocial purposes, and deliberately leads to harmful or immoral results (Cropley et al., 2008, 2010; Harris et al., 2013; Qiao et al., 2022).

According to the definition of MC, malevolent creative behaviors have negative consequences on individuals and society. The randomness, surprise, and rule-breaking features of creativity render malevolent behaviors unpredictable (Gill et al., 2013; Gino and Wiltermuth, 2014; Wang, 2019). Moreover, compared with uncreative unethical behaviors, creative ones are considered less immoral; thus, they are punished less severely (Wiltermuth et al., 2017). This implies that MC increases the success rate of malevolent behavior and makes it easier for implementers to escape punishment. Although MC is usually marked by creative criminal or terrorism activities (Cropley et al., 2008; Gill et al., 2013), it can be observed in the general population (Hao et al., 2020; Perchtold-Stefan et al., 2021), such as framing other colleagues creatively to avoid punishment. These MC behaviors may not be considered crimes but still hurt others’ personal interests. Most people are more likely to encounter the outcomes of MC from ordinary people than from criminals or terrorists. Additionally, noncriminal malevolent creative behavior may escalate into criminal malevolent creative behavior in the future. Therefore, based on the findings from studies on criminals or terrorism, researchers have tried to understand MC in the general population. Several factors that are positively related to MC have been explored, including individual aggression (Lee and Dow, 2011; Harris and Reiter-Palmon, 2015), antagonism (Perchtold-Stefan et al., 2021), lower emotional intelligence (Harris et al., 2013), the Dark Triad (Jonason et al., 2017; Gao et al., 2022b), approach motivation (Hao et al., 2020), and anger emotional state (Cheng et al., 2021a,b). While it would be preliminary and important to explore the underlying mechanisms (including neural substrates) of MC idea generation, which helps to develop effective techniques or approaches to manage and prevent malevolent creative behaviors, as a result prevents the negative effects of malevolent creativity on society.

To date, several studies have directly investigated the neural correlates of idea generation in MC. In these studies, the participants were required to generate creative solutions that caused damage. Qiao et al. (2022) reported that the middle occipital gyrus (MOG) is less involved (i.e., weaker activation and less coupling with other regions) in the generation of malevolent creative ideas, which might reflect the inhibition of moral judgment. These results suggest that a decline in moral criteria supports the emergence of immoral ideas. Another study observed decreased efficiency within the default networks (i.e., the superior frontal gyrus and middle temporal gyrus) in MC idea generation, which might imply reduced social cognition toward victims (Gao et al., 2022a). In addition, lower functional connectivity strength in the postcentral gyrus (POG) may be related to weakened emotional perception (Gao et al., 2022a), while malevolent ideation may be weakened after enhancing activity in the right POG (Gao et al., 2023). Perchtold-Stefan et al. (2023b) used an EEG device to investigate task-related power changes in the alpha band during the generation of malevolent ideas. The results showed that individuals with higher MC performance had increased prefrontal alpha power in the early stage (indicating conceptual expansion from prosocial to antisocial) and increased temporal alpha power in the middle stage (indicating inhibition of dominant but normal ideas; Perchtold-Stefan et al., 2023b). Furthermore, researchers have noticed gender differences in the neural substrates underlying MC. Although females and males have similar levels of MC performance, females tend to generate malevolent creative ideas through semantic memory retrieval and the recombination of social information, whereas males rely more on motor-related imagery (Perchtold-Stefan et al., 2023a).

However, MC is not a one-dimensional framework. Researchers have proposed a possible classification of MC behaviors in daily life, namely hurting people, lying, and playing tricks (Hao et al., 2016). These MC behaviors refer to the generation of creative solutions that can cause damage, but the way in which they cause damage is different, as follows: (1) hurting people indicates obtaining an unfair advantage by harming others physically or mentally (e.g., attacking enemies with new weapons); (2) lying is concerned with telling a lie, concealing, and framing (e.g., designing a novel scam); and (3) playing tricks is used to design a creative prank or practical joke (e.g., setting up a novel trick). According to this definition, hurting people is more dangerous than the other two kinds of MC (Hao et al., 2016), while lying and playing tricks can cause substantial financial or reputation losses. Given that hurting people has been the main focus of the aforementioned neuroscience MC studies, to further understand the neural portrait of MC, the neural substrates (brain activation and cross-regional neural coupling) that underlie the generation of various malevolent creative ideas deserve exploration. Therefore, this study aimed to reveal the similarities and distinctions between the neural substrates underlying idea generation in the three kinds of MC.

Although different kinds of MC vary greatly, they all involve generating creative ideas. Thus, neural responses in the creativity-related regions may be observed in all kinds of MC. According to previous studies, the executive control network (ECN; e.g., the middle frontal gyrus) and default network [DMN; e.g., the right temporal–parietal junction (rTPJ) and precuneus] play important roles in the creative process (Fink et al., 2010; Wu et al., 2015; Beaty et al., 2016, 2017; Zhu et al., 2017). Specifically, the executive control network is related to working memory and task-set switching, which are necessary for creative idea generation (Beaty et al., 2016; Zhu et al., 2017), whereas the DMN is involved in memory retrieval (e.g., rTPJ) and information integration (e.g., angular gyrus (AG)] in creativity (Fink et al., 2010; Zhu et al., 2017). Furthermore, the executive control network cooperates with the default network by inhibiting prominent responses elicited by memory retrieval (Beaty et al., 2017). Moreover, MC is characterized by damaging and breaking social norms; thus, all kinds of MC are related to antisocial behavior and morality. Consistent with this, a previous study found that individuals who were less concerned about moral values tended to have higher MC levels (Kapoor and Kaufman, 2022a). At the neural level, antisocial behavior is related to the dysfunction of the right temporal lobe and reduction of the gray matter volume in the left frontopolar cortex (lFPC) and right POG (rPOG; Fumagalli and Priori, 2012; Bertsch et al., 2013; Aoki et al., 2014). Individuals tend to break reciprocal norms when the right dorsolateral prefrontal cortex (rDLPFC) is inhibited (Knoch et al., 2006). Accordingly, these findings suggest that all kinds of malevolent creative idea generation may be involved in the neural responses of the bilateral PFC and right temporal lobe.

Regardless of their similarities, different kinds of MC differ in specific features. Hurting people is directly related to aggression (Hao et al., 2016). Studies showed that increased physical aggression is accompanied by decreased neural activation or PFC impairment, which may reflect a deficiency in inhibition (Soloff et al., 2003; Nelson and Trainor, 2007). Aggressive individuals exhibit abnormal neural activation in the right precuneus (Wong et al., 2019). Moreover, structural and functional reductions in the AG and superior temporal gyrus are associated with increased hurting behaviors (Raine, 2019). In addition to the PFC, the temporal lobe also plays an important role in generating ideas that hurt people. Lying is characterized by creative and dishonest behavior. Previous research revealed that the frontal (i.e., DLPFC) and temporal regions are involved in dishonest behavior (Lisofsky et al., 2014; Sun et al., 2015; Yin et al., 2016; Ofen et al., 2017). This suggests that cognitive control and perspective taking play essential roles in cheating others (Lisofsky et al., 2014; Ofen et al., 2017). Additionally, the right frontopolar cortex (rFPC) is involved in well rehearsed lies (Ganis et al., 2003; Abe, 2011). Given that creative lies are usually more subtle and complex, rFPC may be involved in weaving malevolent creative lies. Playing tricks is typically used to create fun. Studies demonstrated increased activity in the left inferior frontal gyrus (lIFG) during humor detection (Moran et al., 2004). The theory-of-mind network (i.e., the middle temporal gyrus) is more involved in point-to-other jokes (Feng et al., 2014). Brain regions associated with humor and jokes may overlap with those associated with generating creative tricks.

This study aimed to reveal similar and distinct neural correlates of the three kinds of MC idea generation using a one-way factorial design, with TASK (tasks of hurting people, lying, and playing tricks) as the within-subject factor. The participants were required to complete the following three MC tasks: (1) hurting people; (2) lying; and (3) playing tricks. They were instructed to generate different creative malevolent ideas using the corresponding kind of MC. For example, the participants were only allowed to generate creative methods to cause physical or mental damage during the hurting people task (HPT). Neural activity during each task was recorded using functional near-infrared spectroscopy (fNIRS), which has several advantages, such as (1) higher tolerance of body movement (Schecklmann et al., 2010); (2) higher ecological validity; and (3) greater economy. The common cerebral regions involved in all MC tasks were identified by comparing the neural activation and cross-regional neural coupling of all MC tasks with those at baseline. Subsequently, neural activation and coupling were compared among all kinds of MC to reveal their specific neural correlates. The hypotheses (Hs) were as follows: H1, neural responses in brain regions such as the lFPC, middle frontal gyrus, rPOG, and right MOG (rMOG) would be observed in three kinds of MC; H2a, the hurting people task is more associated with the right precuneus, AG, and superior temporal gyrus; H2b, the lying task (LT) is more associated with the DLPFC and rFPC; and H2c, the playing tricks task (PTT) is more associated with the lIFG and middle temporal gyrus. Moreover, previous studies found that the Dark Triad of personality, moral personality, general creativity potential, and MC potential can affect MC performance (Jonason et al., 2017; Gao et al., 2022b; Kapoor and Kaufman, 2022b; Perchtold-Stefan et al., 2023a). Therefore, we also measured these indices using a series of scales and tested whether these variables were distinctively related to the neural substrates of the different kinds of MC idea generation.

## Materials and Methods

### Participants

A total of 40 participants (mean age = 21.30 ± 2.23 years; 32 females; college students) participated in the study (2 participants were excluded because of poor signal; for details, see the subsection fNIRS data preprocessing). An a priori power analysis showed that the sufficient sample size to obtain reliable results was 36 (1 – β = 0.90, α = 0.05, effect size f = 0.25; Chow et al., 2017), which is comparable to previous neuroscientific creativity studies (Takeuchi et al., 2019; Tempest and Radel, 2019). All the participants were right handed and had normal or corrected-to-normal visual acuity. None of the patients had any history of mental or neurologic illness. Written informed consent was obtained from all participants before the experiment. Each participant received compensation of ¥55. The study procedure was approved by the University Committee on Human Research Protection of the East China Normal University (Code: HR 039-2017). To avoid misrepresentation, the sample in the current study was collected independently and did not overlap with any published studies.

### Experimental procedure

Upon arrival, each participant was asked to sit in front of a laptop table. The experimenter introduced the experimental procedure to the participants, prepared the fNIRS device, and told the participants to be ready. The initial scan session lasted for 30 s and served as the baseline, during which the participants were required to close their eyes, remain still, and relax. The task session consisted of three blocks (10 trials per block). The participants were required to solve 10 hurting people, lying, or playing tricks tasks, respectively, during each block. The sequence of the blocks was counterbalanced among the participants.

**Figure 1.**
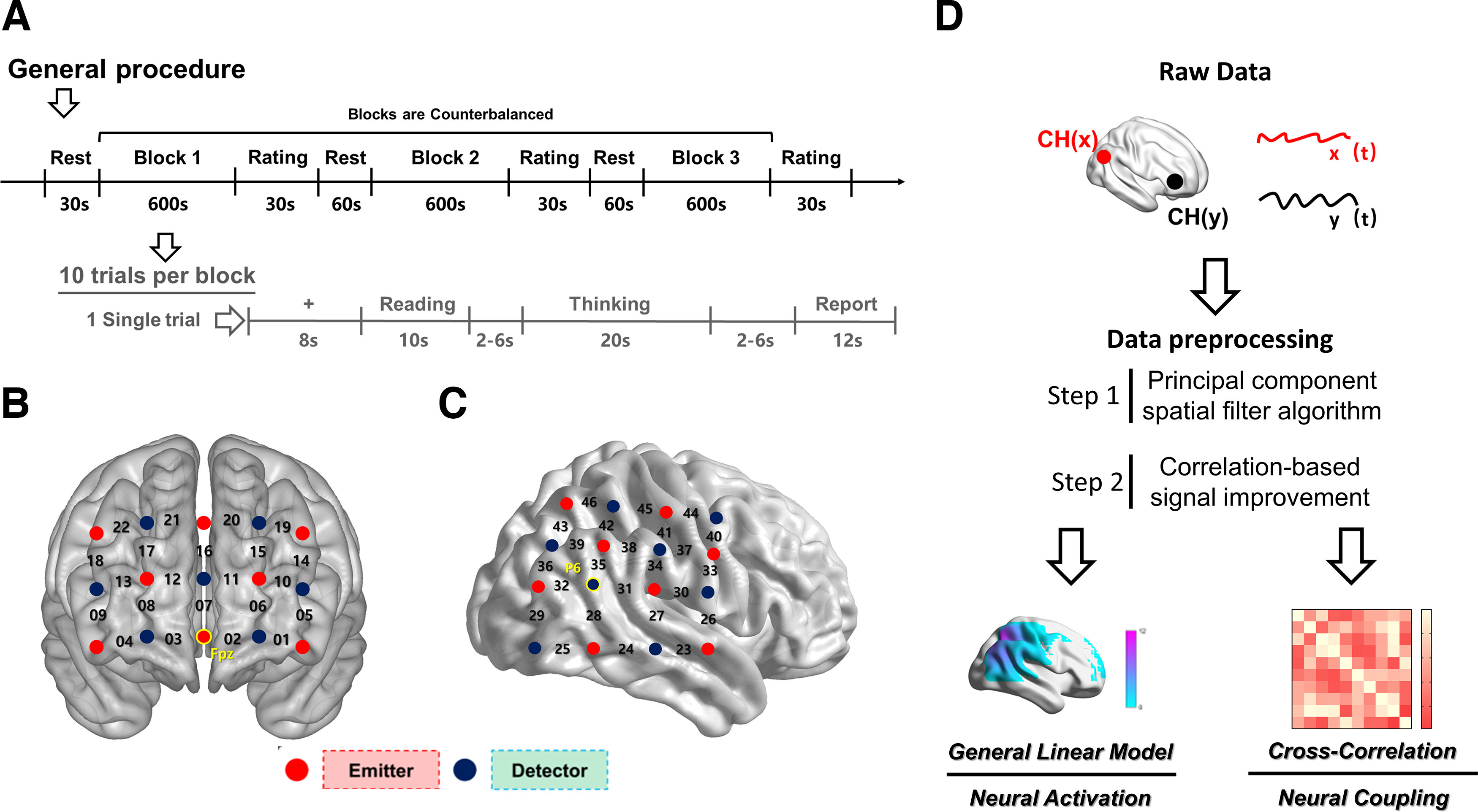
Experimental design in the study. ***A***, Experimental procedure. Rest, 30 s resting state session; rating, 30 s self-rating on anxiety, pleasure, benevolence and malevolence; the task sequence was counterbalanced among participants. ***B***, Optode probe set on the prefrontal cortex. ***C***, Optode probe set on the right temporal regions. ***D***, The data preprocessing and analysis procedure.

Each trial successively consisted of an 8 s fixation session, a 10 s task-reading session, a 20 s thinking session, and a 12 s reporting session. The participants verbally reported their most creative responses in the reporting session (only one response was allowed for each trial). Two jitters (blank screen, 2–6 s) were set between the latter three sessions (Fig. 1*A*).

Immediately after each block, the participants rated their anxiety, pleasure, and benevolence and malevolence of tasks on a 7-point Likert scale ranging from 1 (not at all) to 7 (strongly). The ratings of the malevolence and benevolence of tasks were designed to verify the effectiveness of the tasks (see details in the subsection Experimental tasks). There was a 1 min resting session after each block. Eventually, the participants were asked to complete a series of scales right after they finished the whole experiment (see details in the subsection Postexperimental scales).

### Experimental tasks

The hurting people tasks, lying tasks, and playing tricks tasks were adopted from the previous study. It showed that there were no significant differences in the aspects of difficulty, fatigue, involvement, and familiarity among these three MC tasks (Cheng et al., 2021b).

#### Hurting people task

This task was adapted from a realistic presented problem, which requires individuals to generate a creative solution for an open-ended realistic problem (Agnoli et al., 2016; Runco et al., 2016; Xue et al., 2018). Each HPT problem was expressed using words limited to 38–42 Chinese characters. The participants were explicitly required to solve the problem by causing physical or mental damage in novel ways (e.g., Hong encounters a tennis master in the final game, which makes it difficult for her to win. Please generate a novel way for Hong to injure her opponent “accidentally”). It should be noted that the participants could generate several ideas; however, they only needed to report the idea they considered the most creative. The participants were asked to rate the intelligibility, malevolence, and benevolence of the 10 HPT problems on a Likert scale ranging from 1 (very low) to 7 (very high). These 10 HPT problems showed the following: (1) intelligibility was all >6; (2) malevolence was all >5; and (3) benevolence was all <3.

#### Lying task

The procedure for developing this task was similar to that for the hurting people task. The participants were required to solve the problem by lying, concealing, and framing in novel ways (e.g., Ming has stolen public donations for private use but does not want to be caught. Please generate a novel way for Ming to conceal this thing). The intelligibility, malevolence, and benevolence of 10 LT problems were >6, >5, and <3, respectively.

#### Playing tricks task

The procedure for developing this task was similar to that for the hurting people task. The participants were required to solve the problem using a creative prank or practical joke (e.g., A head teacher who is very strict toward Jie intends to host an open class. Please generate a novel way for Jie to ruin the open class without being caught). The intelligibility, malevolence, and benevolence of the 10 PTT problems were >6, >5, and <3, respectively.

### Assessment of performance during the hurting people, lying, and playing tricks tasks

Performance during the hurting people, lying, and playing tricks tasks was assessed by evaluating the originality and harmfulness of the ideas (Runco and Okuda, 1991; Perchtold-Stefan et al., 2021, 2022; Gao et al., 2022a; Qiao et al., 2022). As only one idea was allowed for each task, idea fluency or uniqueness was not assessed (Lu et al., 2019a). Thus, a widely used subjective method was used to assess originality and harmfulness of the ideas (Beaty et al., 2018; Xue et al., 2018; Lu et al., 2019b). Five trained raters (with at least 3 years of experience in creativity research) independently scored originality and harmfulness of the ideas on a 5-point Likert scale (originality: 1 = not original at all, 5 = highly original; harmfulness: 1 = not harmful at all, 5 = highly harmful). The interrater agreement was satisfactory [internal consistency coefficient (ICC); originality: ICC_[hurting people task]_ = 0.79, ICC_[lying task]_ = 0.70, ICC_[playing tricks task]_ = 0.72; harmfulness: ICC_[hurting people task]_ = 0.80, ICC_[lying task]_ = 0.74, ICC_[playing tricks task]_ = 0.70). The originality and harmfulness scores for each idea were calculated by averaging the scores of all the raters. The final scores of the participants were calculated by averaging the scores for all the ideas.

### Postexperimental tests

The Runco Ideational Behavior Scale (RIBS; e.g., I have a lot of ideas about stories and poetry; Runco et al., 2016) was used to evaluate the participants’ general creativity potential. The RIBS contains 19 items rated on a 5-point Likert scale ranging from 0 (never) to 4 (approximately every day). The internal consistency reliability was satisfactory (Cronbach’s α = 0.88). The Malevolent Creativity Behavior Scale (MCBS; e.g., I have thought of some new methods to punish people who do something wrong; Hao et al., 2016) was used to evaluate the participants’ MC potential. The MCBS contains 13 items rated on a 5-point Likert scale ranging from 1 (never) to 5 (always). The internal consistency reliability was satisfactory (Cronbach’s α = 0.84). The Honesty-Humility Inventory (Ho-Hu; e.g., If I knew I would never get caught, I would want to steal a million yuan; Lee and Ashton, 2004) was used to evaluate the participants’ moral personalities. The Ho-Hu contains 20 items rated on a 5-point Likert scale ranging from 1 (strongly disagree) to 5 (strongly agree). The internal consistency reliability was satisfactory (Cronbach’s α = 0.78). The Chinese version of the Dirty Dozen (DD12; e.g., I am used to getting my own way by manipulating others; Geng et al., 2015) was used to evaluate the participants’ Dark Triad personality traits. The DD12 contains 12 items rated on a 7-point Likert scale ranging from 1 (strongly disagree) to 7 (strongly agree). The internal consistency reliability of the DD12 was unsatisfactory in this study (Cronbach’s α = 0.68); therefore, this scale was not included in the subsequent analysis.

### fNIRS data acquisition

An fNIRS device (model ETG-7100, Hitachi Medical) was used to record the concentrations of oxyhemoglobin (HbO) and deoxyhemoglobin (HbR) during the experiment. The absorption of near-infrared light (wavelengths, 695 and 830 nm) was assessed at a sampling rate of 10 Hz. The bilateral PFC and right temporal region were selected as ROIs in this study. A 3 × 5 probe set [eight emitters and seven detectors, 3 cm optode separation, forming 22 measurement channels (CHs); Fig. 1*B*] was placed on the bilateral PFC, and one 4 × 4 probe set (eight emitters and eight detectors, 3 cm optode separation, forming 24 measurement channels; Fig. 1*C*) was placed on the right temporal region. According to the 10–20 system, the lowest row of the 3 × 5 probe set was aligned with the Fp1–Fp2 line, and optode “A” was positioned on the frontal pole midline point (Fpz; Sai et al., 2014). In the meantime, the middle probe column of the 3 × 5 probe set was aligned along the sagittal reference curve (Fig. 1*B*). The 4 × 4 probe set was aligned with the sagittal reference curve, and optode “B” was positioned on P6 (Fig. 1*C*). The virtual registration method was used to establish the correspondence between channels and measurement points (brain regions) in the cortex (Singh et al., 2005; Tsuzuki et al., 2007).

### fNIRS data preprocessing

The principal component spatial filter algorithm was used to remove the global components of the raw fNIRS data, whereas the correlation-based signal improvement method was used to remove motion artifacts (Cui et al., 2012; Pan et al., 2018; Fig. 1*D*). Considering that the HbO and HbR signals were negatively correlated after using the correlation-based signal improvement method (the corrected HbR is solely the corrected HbO multiplied by a negative coefficient; Cui et al., 2010), the subsequent analysis mainly focused on HbO signals.

Channels with poor signals were determined by visually checking the NIRS time course plot. The channel with a considerably higher variance than other channels of the same participant was identified as a poor channel (e.g., the variances of normal channels were 0.5∼0.8, but those of poor channels were 10∼30). Participants with >11 poor channels (25% of the total channels) were excluded from the subsequent analysis.

### Data analysis of neural activation

The NIRS Statistical Parametric Mapping (NIRS_SPM) package was used to estimate individual neural activation in this study (Jang et al., 2009; Ye et al., 2009). The hemodynamic response function (hrf) low-pass filtering and wavelet minimum description length detrending algorithms were selected as required by the NIRS_SPM. Neural activation during the entire thinking stage (0–20 s) was estimated using a general linear model (Fig. 1*D*). First, a reference wave was set for each channel for all conditions (baseline, hurting people task, lying task, and playing tricks task) to represent the theoretical variations in HbO signals induced by the experimental stimulus. Then, a regression analysis containing theoretical HbO signal variations and real HbO signal variations during the task period (baseline and thinking stage of hurting people, lying, and playing tricks tasks) was performed for each channel. β Values, which indicate variation in neural activation, were obtained as regression coefficients for all channels under different conditions. Furthermore, the β increment was calculated by subtracting the baseline β value from the thinking-session β value for the three tasks.

Primarily, one-sample *t* tests using 0 as the test value (baseline) were performed on the β increments of each task. All *p*-values were corrected using the false discovery rate (FDR) correction method (corrected α level = 0.05). The channels that were significant in all three tasks were marked as common activated or common deactivated.

Next, the β increments were *z*-scores transformed channel by channel across the participants. A total of 46 one-way repeated-measures ANOVAs, using TASK as the within-subject factor, were performed on the β increments. All *p*-values were corrected using the FDR method (corrected α level, 0.05). Subsequent *post hoc* tests were corrected using Bonferroni correction when necessary.

A nonparametric permutation test was performed on significant channels (Maris and Oostenveld, 2007; Zheng et al., 2020). The procedure was as follows: (1) within each channel, we randomly sampled the data from three MC tasks to reconstruct three new subsets; (2) one-way repeated-measures ANOVAs using three new subsets as within-subject factors were conducted to calculate a new *F* value; (3) we repeated subsets 1 and 2 5000 times to establish a distribution of *F* values (i.e., 5000 one-way repeated-measures ANOVAs on 5000 3 subsets); and (4) if the actual *F* value was >95% of the *F* values in the distribution (permutation threshold, *p*<0.05), differences among the three tasks were considered significant.

Linear regression was performed to quantify the relationship between β increments from all the channels and task performance. Before the formal linear regression procedure, several Pearson correlation analyses were performed on the behavioral performance and β increments of each channel. Only the channels that were significantly correlated with behavioral performance (*p*<0.05, uncorrected) were introduced into the subsequent analysis as predictors (Xie et al., 2022). In this study, linear regression was performed on every behavioral performance (with β increments as predictors). In addition, the relationships between the postexperimental scales and β increments were examined using linear regression (with postexperimental scales as dependent variables and β increments as predictors).

### Data analysis of neural coupling

Neural coupling was introduced to measure the functional connectivity between different cerebral regions in this study. Cross-correlations were used to assess the neural coupling (NC) between time series HbO concentrations from different cerebral regions during different MC tasks, which were suitable for evaluating the covariation of two signals over time (Ward et al., 2018; Liu et al., 2021; Fig. 1*D*). The NC values (i.e., cross-correlation coefficients) were converted using Fisher’s *z*-transformation. The NC increment was calculated by subtracting the baseline NC value from the NC values of the three tasks.

Initially, there were 2116 CH combinations (46 × 46 CHs). After excluding 1081 redundant CH combinations (equal CH combinations or CH combinations of a single CH; i.e., CH1–CH1), only 1035 valid CH combinations were finally entered into the subsequent analyses. Likewise, several one-sample *t* tests using 0 as the test value (baseline) were performed on the NC increments of each task. All *p*-values were corrected using the FDR method (corrected α level, 0.05). The NC increments of CH combinations that were significantly higher than baseline in all the three tasks were marked as the common increased NC increments. Next, several one-way repeated-measures ANOVAs, using TASK as the within-subject factor, were performed on the NC increment for each CH combination. The *p*-values were corrected using the FDR method across all CH combinations (1035 combinations; corrected α level, 0.05). Subsequent *post hoc* tests were corrected using Bonferroni correction when necessary. A nonparametric permutation test was performed on significant NC increments to validate the results (Maris and Oostenveld, 2007; Zheng et al., 2020).

Linear regression analysis, similar to the β increment, was performed to quantify the relationship between the NC increments of all the CH combinations and task performance. Considering that there were 1035 CH combinations, only the CH combinations that were significantly correlated with behavioral performance (*p*<0.005, uncorrected) were introduced into the subsequent linear regression as predictors (Xie et al., 2022). The remaining procedure was the same as that used for neural activation (see details in the subsection Data analysis of neural activation). Additionally, linear regression with the same procedure was used to examine the relationship between postexperimental scales and NC increments.

### Data availability

The code used in this study is available from the corresponding author on request.

## Results

### Behavioral indices

One-way repeated-measures ANOVAs, using TASK as the within-subject factor, were performed for anxiety, pleasure, benevolence, and malevolence. The results showed that the main effect of TASK on anxiety was significant (*F*_(2,78)_ = 4.17, *p *=* *0.019, *η_p_^2^* = 0.10). The *post hoc* test (Bonferroni corrected) revealed that the participants experienced significantly more anxiety during the hurting people task than during the playing tricks task (*p *=* *0.043). The results also showed a significant main effect of TASK on pleasure (*F*_(2,78)_ = 5.20, *p *=* *0.008, *η_p_^2^* = 0.12). The *post hoc* test revealed that the participants significantly experienced more pleasure during the playing tricks task than during the lying task (*p *=* *0.004). Detailed descriptive data are presented in Tables 1 and 2.

**Table 1:** The results of one-way repeated-measures ANOVAs with TASK as the within-subject factor on the anxiety, pleasure, benevolence, and malevolence

	HPT_(mean ± SD)_	LT_(mean ± SD)_	PTT_(mean ± SD)_	*F*_(2,78)_	*p*	η*_p_^2^*	*Post hoc* test^*a*^
Anxiety	5.21 ± 1.44	4.97 ± 1.49	4.59 ± 1.58	4.17	0.019	0.10	HPT > PTT*
Pleasure	2.28 ± 1.40	1.86 ± 0.76	2.46 ± 1.28	5.20	0.008	0.12	PTT > LT**
Benevolence	1.45 ± 0.67	1.70 ± 0.87	1.79 ± 0.76	5.17	0.008	0.12	PTT > HPT**
Malevolence	6.68 ± 0.46	5.95 ± 0.88	5.84 ± 1.17	13.42	0.000	0.26	HPT > LT*** HPT > PTT***

*p <* *0.05; **p <* *0.01; ***p <* *0.001.

*^a^Post hoc* tests were Bonferroni corrected.

**Table 2:** The results of one-way repeated-measures ANOVAs with TASK as the within-subject factor on the NC increments

CH combination^*a*^	HPT_(mean ± SD)_	LT_(mean ± SD)_	PTT_(mean ± SD)_	*F*	*p*_(FDR)_	η*_p_^2^*	*Post hoc* test^*b*^
CH8–CH21	0.00 ± 0.86	−0.53 ± 0.91	−0.04 ± 1.11	9.49_(2,74)_	0.037	0.20	LT < HPT*** LT < PTT**
CH12–CH46	−0.20 ± 0.66	−0.51 ± 0.67	0.07 ± 0.95	10.56_(2,74)_	0.048	0.22	LT < HPT** LT < PTT***
CH13–CH14	0.16 ± 1.36	0.35 ± 1.21	−0.25 ± 1.05	9.56_(2, 72)_	0.043	0.21	PTT < HPT* PTT < LT***
CH17–CH38	−0.37 ± 0.97	0.33 ± 0.96	0.24 ± 1.01	9.66_(2,74)_	0.048	0.21	HPT < LT** HPT < PTT**

*p <* *0.05; **p <* *0.01; ***p <* *0.001.

*^a^*Only CH combinations that survived the FDR correction are listed.

*^b^*The *post hoc* tests were Bonferroni corrected.

The main effect of TASK on benevolence was significant (*F*_(2,78)_ = 5.17, *p *=* *0.008, *η_p_^2^* = 0.12). The *post hoc* test (Bonferroni corrected) revealed that the playing tricks task contained significantly more benevolence than the hurting people task (*p *=* *0.005). A one-sample *t* test (test value = 3) revealed that the benevolence of the hurting people (*t*_(39)_ = −14.70, *p *< 0.001, Cohen’s *d = *2.32), lying (*t*_(39)_ = −9.40, *p *< 0.001, Cohen’s *d = *1.49), and playing tricks tasks (*t*_(39)_ = −10.07, *p *< 0.001, Cohen’s *d = *1.59) were significantly <3. The main effect of TASK was also significant for malevolence (*F*_(2,78)_ = 13.42, *p *< 0.001, *η_p_^2^* = 0.26). The *post hoc* test (Bonferroni corrected) revealed that the hurting people task contained more malevolence than the lying (*p *<* *0.001) and playing tricks tasks (*p *<* *0.001). A one-sample *t* test (test value = 5) revealed that the malevolence of the hurting people task (*t*_(39)_ = 23.24, *p *< 0.001, Cohen’s *d = *3.68), lying task (*t*_(39)_ = 6.83, *p *< 0.001, Cohen’s *d = *1.08), and playing tricks task (*t*_(39)_ = 4.54, *p *< 0.001, Cohen’s *d = *0.72) was significantly >5. These results partially confirm the effectiveness of the three tasks. Detailed descriptive data are presented in Table 1.

### β increments

One-sample *t* tests using 0 as the test value (baseline) were performed on the β increments of each task among all the CHs (see Table 3, see details). After FDR correction (*p *<* *0.05, a total of 46 channels), the results revealed that the bilateral FPC (CH2, CH3, and CH4), right middle frontal gyrus (CH9), right AG (rAG; CH39), and right precuneus (CH43 and CH46) were more activated than baseline; the rDLPFC (CH16), right middle temporal gyrus (CH23), right superior temporal gyrus (CH27), rPOG (CH30), and right supramarginal gyrus (rSMG; CH34) were less activated than baseline.

**Table 3:** Results of one-sample *t* test on β increment

Channel	*t*-Values^*a*^			Region
	HPT	LT	PTT	
2	3.42**	4.16***	3.24*	Left FPC
3	5.72***	5.91***	4.67***	Right FPC
4	4.48***	6.84***	3.51**	Right FPC
9	2.90*	3.10*	2.38*	Right MFG
16	−2.65*	−2.60*	−2.75*	Right DLPFC
23	−3.06*	−3.91**	−4.23**	Right MTG
27	−5.59***	−3.11*	−3.61**	Right STG
30	−3.66**	−4.11**	−3.80*	Right POG
34	−3.46**	−4.27**	−3.31**	Right SMG
39	5.19***	5.00***	5.56***	Right AG
43	5.70***	5.73***	5.63***	Right precuneus
46	3.58**	3.95**	4.14**	Right precuneus

*Post hoc* tests were Bonferroni corrected. MFG, Middle frontal gyrus; MTG, middle temporal gyrus; STG, superior temporal gyrus.

*p <* *0.05; **p <* *0.01; ***p <* *0.001.

*^a^*Only channels that were significantly different from baseline in all tasks are listed.

**Figure 2.**
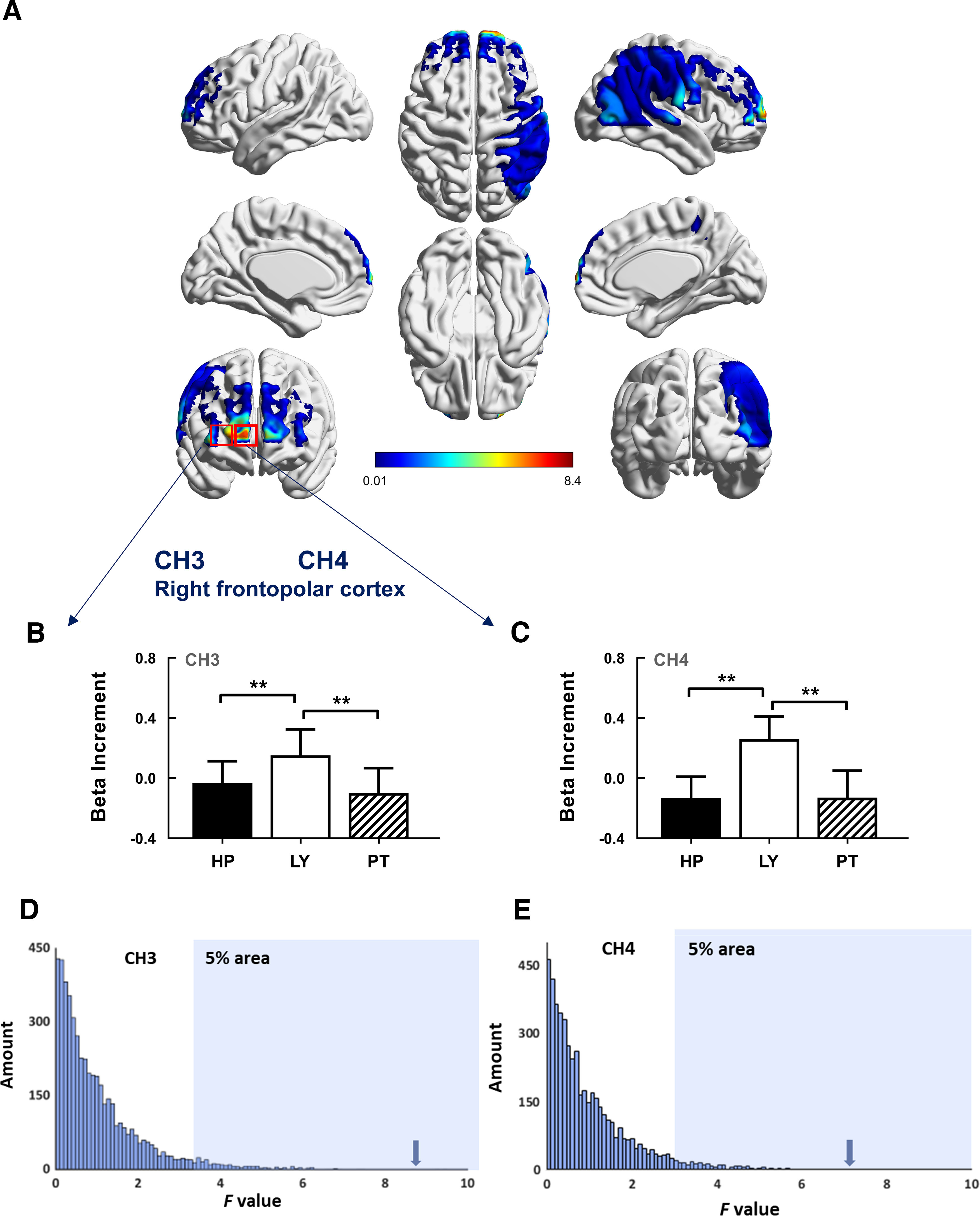
Results on β increment (FDR corrected). HP, Hurting people; LY, lying; PT, playing trick. ***A***, The full views of the main effect of TASK on the β increment of all CHs. The red rectangle indicates that the CHs have significant main effect (CH3 and CH4). ***B***, The amplitude of the β increment of CH3. *p <* *0.05, **p <* *0.01, ***p <* *0.001. ***C***, The amplitude of the β increment of CH4. *p <* *0.05, **p <* *0.01, ***p <* *0.001. ***D***, ***E***, The distribution of *F* values from permutation test on CH3/CH4. The arrows denote the location of the actual *F* value.

One-way repeated-measures ANOVAs, using TASK as the within-subject factor, were performed on the β increments of all CHs. Individual data with poor signals were excluded from each CH. After FDR correction (*p *<* *0.05, a total of 46 channels), the results revealed that the main effects of TASK were significant on the β increment at CH3 (*F*_(2,76)_ = 8.40, *p *=* *0.023, *η_p_^2^* = 0.18; Fig. 2*B*) and CH4 (*F*_(2,76)_ = 7.47, *p *=* *0.025, *η_p_^2^* = 0.16; Fig. 2*C*). Both CH3 and CH4 are located in the rFPC. *Post hoc* tests (Bonferroni corrected) showed that the β increment at CH3 was significantly higher during the lying task (mean* *= 0.15, SD = 1.08) than during the hurting people task (mean = –0.05, SD = 0.89, *p *=* *0.009) and playing tricks task (mean = –0.10, SD = 1.03, *p *= 0.002). Moreover, the β increment at CH4 was significantly higher during the lying task (mean* *= 0.26, SD = 0.94) than during the hurting people task (mean = –0.13, SD = 0.89, *p *=* *0.003) and the playing tricks task (mean = –0.13, SD = 1.13, *p *=* *0.006). The permutation test revealed that the *F* values of these results were larger than the 95% empirical distribution (Fig. 2*D*,*E*).

### Neural couplings

One-sample *t* tests, using 0 as the test value (baseline), were performed on the NC increments of each task among all CH combinations (see Table 4, see details). After FDR correction (*p *<* *0.05, a total of 1035 valid channels), the following NC increments were more coupled than baseline: lFPC and left DLPFC (lDLPFC; CH1–CH5), right fusiform and rMOG (CH24–CH25), rAG and rMOG (CH32–CH36), rSMG and rAG (CH35–CH38, CH38–CH39, and CH39–CH42), and rPOG and right precentral gyrus (CH41–CH44 and CH44–CH45).

**Table 4:** Results of one-sample *t* test on NC increment

CH combination	*t*-Values^*a*^			Region
	HPT	LYT	PTT	
CH1–CH5	3.74*	5.55***	3.28*	Left FPC-left DLPFC
CH24–CH25	3.50*	4.98**	4.03*	Right fusiform-rMOG
CH32–CH36	3.91*	7.93***	6.39***	Right AG-rMOG
CH35–CH38	4.00*	3.59*	4.36**	Right AG-right SMG
CH38–CH39	4.18*	6.02***	5.23***	Right SMG-right AG
CH39–CH42	5.60***	4.80**	3.65*	Right AG-right SMG
CH41–CH44	4.23*	3.22*	4.01*	Right POG-right PRG
CH44–CH45	7.19***	5.81***	5.76***	Right PRG-rPOG

The *post hoc* tests were Bonferroni corrected. PRG, Precentral gyrus.

*^a^*Only CH combinations that were significantly different from baseline in all tasks are listed.

*p <* *0.05; **p <* *0.01; ***p <* *0.001.

**Figure 3.**
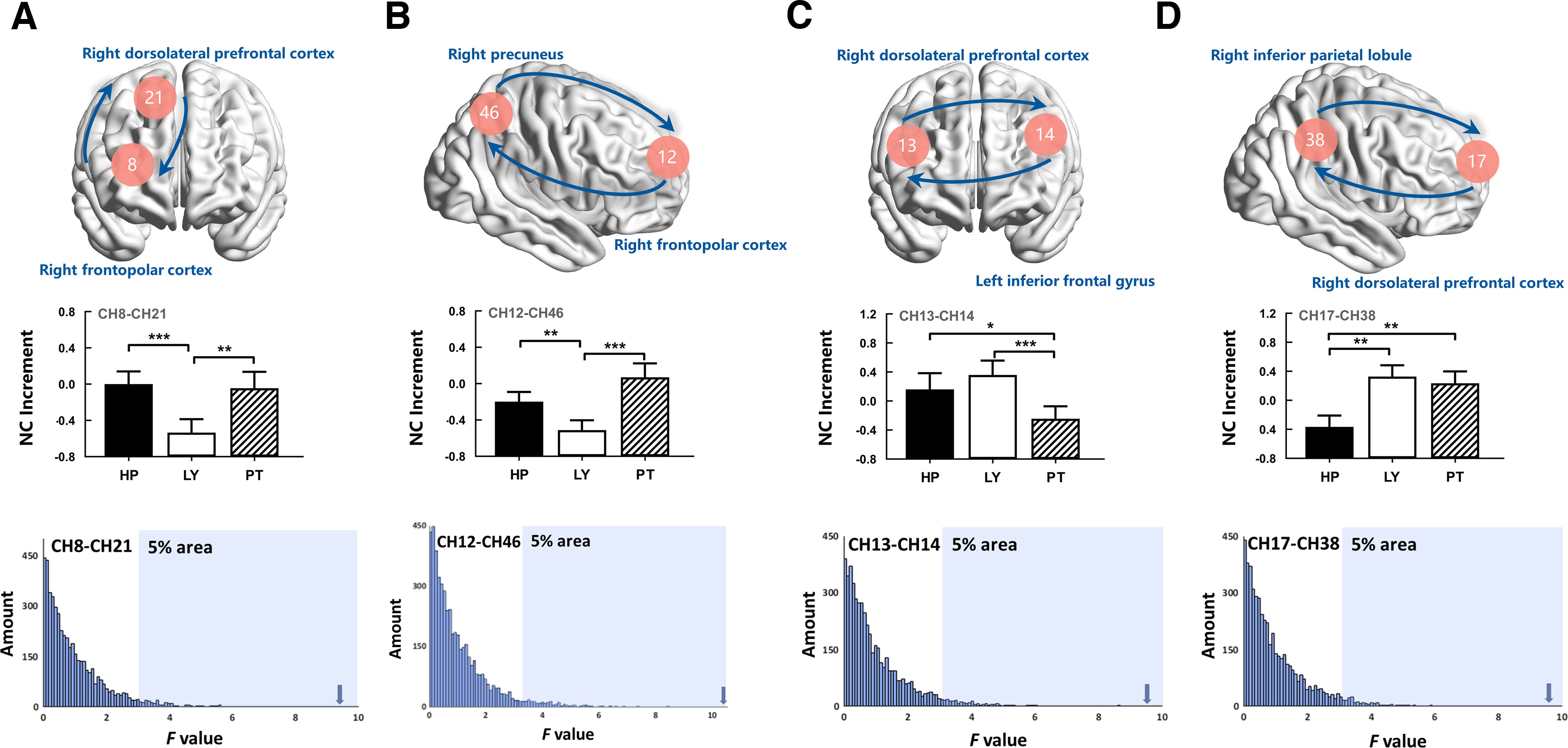
Results on NC increment (FDR corrected). HP, Hurting people; LY, lying; PT, playing trick. ***A–D***, The amplitude of NC increments of CH8–CH21, CH12–CH46, CH13–CH14, and CH17–CH18. The distributions of *F* values from permutation test on these CH combinations. The arrows denote the locations of actual *F* values. *p <* *0.05, **p <* *0.01, ***p <* *0.001.

Several one-way repeated-measures ANOVAs, using TASK as the within-subject factor, were performed for the NC increments of the valid CH combinations. Individual data with poor signals were excluded from each CH combination. After FDR correction (*p *<* *0.05; a total of 1035 valid CH combinations), the results revealed that the main effects of TASK on the NC increments were significant for the following CH combinations: CH8–CH21, CH12–CH46, CH13–CH14, CH17–CH38, and CH27–CH31. Because CH27 and CH31 were in the same region, this result was not included in further analyses (Fig. 3). These CH combinations refer to the rFPC, rDLPFC, right precuneus, lDLPFC, and right inferior parietal lobule (rIPL).

Specifically, *post hoc* tests (Bonferroni corrected) revealed that the NC increment of CH8–CH21 (rFPC–rDLPFC) was significantly lower during the lying task than during the hurting people (*p *<* *0.001) and playing tricks task (*p *=* *0.004; Fig. 3*A*). The NC increment of CH12–CH46 (rFPC-right precuneus) was significantly lower during the lying task than during the hurting people task (*p *=* *0.009) and playing tricks task (*p *<* *0.001; Fig. 3*B*). The NC increment of CH13–CH14 (rDLPFC-lIFG) was significantly lower during the playing tricks task than during the hurting people task (*p *=* *0.019) and lying task (*p *< 0.001; Fig. 3*C*). The NC increment of CH17–CH38 (rDLPFC–rIPL) was significantly lower during the hurting people task than during the lying (*p *=* *0.002) and playing tricks task (*p *=* *0.002; Fig. 3*D*). Detailed descriptive data are listed in Table 2. The permutation test revealed that the *F* values of these results were >95% of the empirical distribution.

Considering that the levels of anxiety and pleasure were significantly different across the three tasks, the linear mixed model was introduced to control for the potential effect of anxiety and pleasure on the results of β increments and neural couplings. The ANOVA results remained significant even after controlling for anxiety and pleasure. This suggests that these neural results are independent of anxiety and pleasure.

### Linear regression analysis

#### The originality and harmfulness of MC tasks

Linear regression analysis was used to quantify the relationship between neural results (β increments and neural couplings) under different conditions and task performances (i.e., originality and harmfulness of the hurting people, lying, and playing tricks tasks). The results revealed that for the hurting people task, harmfulness was negatively predicted by the activation of rPOG (CH30; *b* = −19.70; 95% CI = 0.15, 35.58; *F *=* *6.03; *p *=* *0.005) and originality was negatively predicted by the neural coupling of lDLPFC−rPOG (CH5−CH35; *b* = −1.65; 95% CI = −2.78, −0.53; *F *=* *10.09; *p* < 0.001). For the lying task, originality was positively predicted by the neural coupling between rFPC and rAG (CH12−CH35; *b *=* *1.60; 95% CI = 0.47, 2.73; *F *=* *5.50, *p* < 0.001). For the playing tricks task, harmfulness was negatively predicted by the activation of rDLPFC (CH21; *b* = −20.94; 95% CI = −40.85, −1.03; *F *=* *4.53; *p *=* *0.040), and originality was negatively predicted by the neural coupling between the right superior temporal gyrus and rSMG (CH26–CH34; *b* = −0.80; 95% CI = −1.51, −0.08; *F *=* *6.78; *p* < 0.001).

#### Postexperimental scales

Linear regression analysis was used to quantify the relationship between the neural activity and the scores of the postexperimental scales. The results revealed that the score of the DD12 (Dark Triad) was negatively predicted by the activation of the rIFG during the hurting people task (CH18; *b* = −42.95; 95% CI = −73.26, −12.63; *F *=* *4.89; *p *=* *0.002). The score of the Ho-Hu (moral personality) was negatively predicted by the activation of the right fusiform gyrus during the hurting people task (CH28; *b* = −85.67; 95% CI = −169.64, −1.70; *F *=* *4.34; *p *=* *0.001) and lying task (CH28; *b* = –0.83.68; 95% CI = −163.41, −3.95; *F *=* *5.60; *p* < 0.001). The score of the RIBS (i.e., general creativity potential) was negatively predicted by the activation of the lDLPFC during the hurting people task (CH5; *b* = −55.63; 95% CI = −109.31, −1.95; *F *=* *8.36; *p *=* *0.001) and lying task (CH5; *b* = −58.01; 95% CI = −115.39, –0.62; *F *=* *4.19; *p *=* *0.048) as well as the neural coupling of rFPC–lDLPFC during the playing tricks task (CH8–CH15; *b* = −3.35; 95% CI = −6.33, –0.37; *F *=* *7.72; *p *=* *0.001). The score of the MCBS (i.e., MC potential) was negatively predicted by the neural coupling of rDLPFC–rPOG during the hurting people task (CH22–CH30; *b* = −1.24; 95% CI = −2.41, –0.07; *F *=* *6.57; *p* < 0.001), the score of the lying task was positively predicted by the neural coupling of lDLPFC–rFPC (CH5–CH8; *b *=* *0.70; 95% CI = 0.11, 1.28; *F *=* *5.85; *p* < 0.001), and the score of the playing tricks task was negatively predicted by the neural coupling of rMOG–rSMG (CH25–CH42; *b* = –0.63; 95% CI = −1.16, –0.09; *F *=* *6.48; *p *=* *0.001).

## Discussion

**Figure 4.**
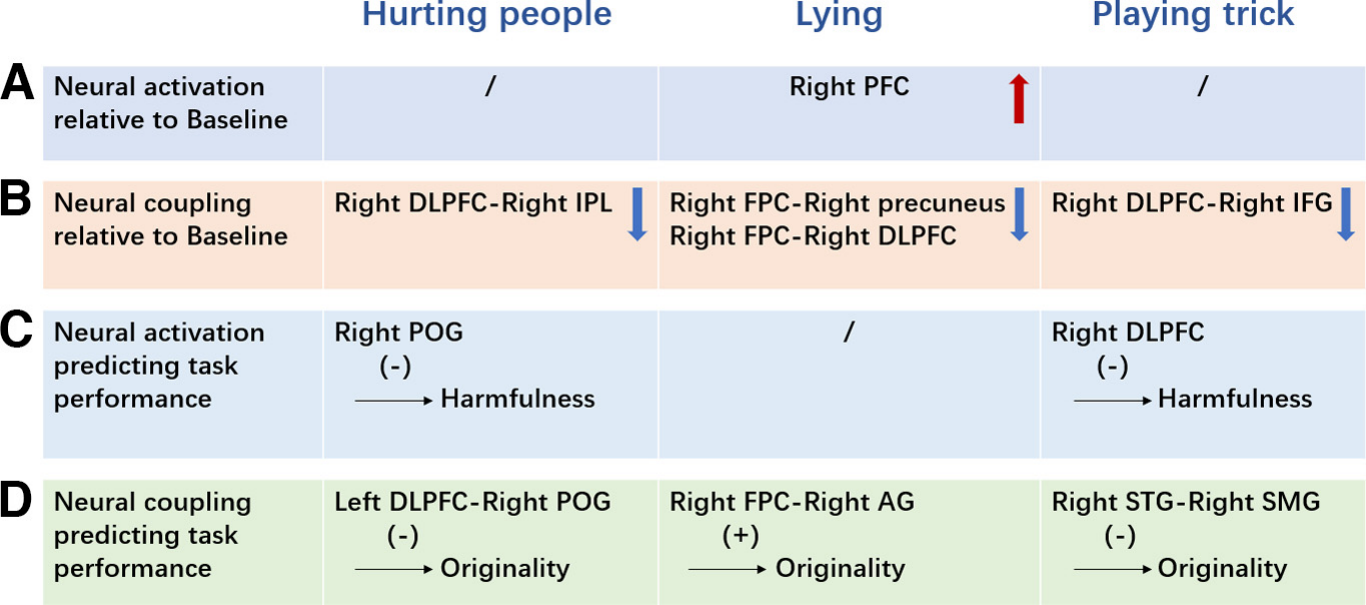
Summary figure of results. STG, Superior temporal gyrus. ***A***, Results of neural activation; the upward arrow means that the neural activation during a certain task was significantly weaker than other tasks (e.g., the neural activation at right FPC during lying task was significantly stronger than during hurting people and playing trick tasks). ***B***, Results of neural coupling; the downward arrow means that the neural coupling during a certain task was significantly weaker than in other tasks (e.g., the neural coupling between right DLPFC and right IPL during a hurting people task was significantly weaker than during lying and playing trick tasks). ***C***, Results of linear regression analysis on neural activation; the symbol (–) means negative prediction (e.g., the neural activation of right DLPFC during playing trick significantly negatively predict the performance of playing trick task). ***D***, Results of linear regression analysis on neural coupling; the symbol (+) means positive prediction (e.g., the neural coupling between right FPC and right AG during lying significantly positively predict the performance of lying task).

This study aimed to reveal similar and distinct neural correlates of the three kinds of malevolent creative idea generation (i.e., hurting people, lying, and playing tricks). The cerebral activity was recorded using an fNIRS device. Several regions of the PFC and temporal lobe were involved in all kinds of MC idea generation, including the bilateral FPC, bilateral DLPFC, and rAG. For each kind, the rFPC during lying task was more activated and less coupled with the rDLPFC and right precuneus than baseline. The rDLPFC was less coupled with the lIFG/rIPL than baseline when playing tricks/hurting people (Fig. 4*A–D*, summary figures).

The results revealed that some regions (including those proposed in H1) were involved in all three kinds of MC. The bilateral FPC, right middle frontal gyrus, and right precuneus were more activated in malevolent creativity, whereas the neural coupling of the lFPC–lDLPFC was stronger than that at baseline. Increased activity in these regions is associated with general creativity (Chen et al., 2015; Green et al., 2015; Wu et al., 2015; Zhu et al., 2017). Moreover, the right middle temporal gyrus and right superior temporal gyrus were less activated, which may reflect higher openness and divergent thinking (Vartanian et al., 2018; Zabelina et al., 2019). These results indicate that individuals attempted to generate creative ideas during all tasks. Furthermore, the rDLPFC, rPOG, and rSMG were less activated than they were at baseline. Reduced activity in these regions involves selfish behavior, such as releasing self-interest motivation (Knoch et al., 2006), less empathy (Seehausen et al., 2016), and more emotional egocentricity (Silani et al., 2013). Additionally, the results showed that the activity of the rAG was higher than that at baseline and more coupled with the rMOG and rSMG. Neural coupling between the right fusiform gyrus and the rMOG, rPOG, and right precentral gyrus was stronger than that at baseline. Previous studies found that the rAG, right fusiform gyrus, and right precentral gyrus are associated with attention reorientation (Hahn et al., 2006; Seghier, 2013; Caspers et al., 2014). The activities of the MOG, rSMG, and rPOG are associated with prosocial behaviors, such as moral judgment (Cheng et al., 2021), inhibiting emotional egocentricity (Silani et al., 2013), and empathy (Seehausen et al., 2016). These results suggest that individuals convert their attention to antisocial task requirements to avoid being affected by prosocial tendencies.

As predicted, we observed distinctions in neural responses among the three kinds of MC idea generation. Specifically, rFPC activation was stronger during the lying task than during the other tasks, which is consistent with H2b. A previous study showed that the bilateral frontal poles (especially the right frontal pole) are associated with theory of mind (ToM; Lewis et al., 2011; Reniers et al., 2014), which is closely related to lying or deception (Poletti et al., 2011; Walczyk et al., 2014; Sai et al., 2021; Walczyk and Cockrell, 2022). Liars rely on ToM to manipulate victims’ beliefs, predict their potential actions, and speculate about their thoughts (Walczyk et al., 2014; Walczyk and Cockrell, 2022). Moreover, rFPC is involved in well rehearsed lies (Ganis et al., 2003; Abe, 2011). Research indicates that the frontopolar region is associated with high-level executive strategies for deception (Ganis et al., 2003; Abe et al., 2006; Lin et al., 2021). These findings emphasize the importance of rFPC in weaving well rehearsed, convincing, and creative lies.

The NC increment in the rFPC–rDLPFC was significantly lower during the lying task than during the hurting people and playing tricks tasks. As stated above, rFPC is involved in well rehearsed lies, and we observed an increase in β increments during the lying task. Research showed that individuals’ honesty increases after neural augmentation of the rDLPFC using transcranial direct current stimulation (Maréchal et al., 2017). In this case, a decrease in the NC of the rFPC–rDLPFC may allow individuals to be dishonest and generate creative lies. In addition, we observed a decrease in the NC of the rFPC–right precuneus when compared with the hurting people and playing tricks tasks. The right precuneus is associated with compassion, a social emotion aroused by personal loss and social deprivation (Immordino-Yang et al., 2009). Accordingly, such a decrease in the rFPC–right precuneus may indicate that personal compassion toward victims is inhibited when generating a creative lie. Linear regression analysis demonstrated that neural coupling between the rFPC and rAG positively predicted the originality of the lying task. The rFPC is related to ToM (Lewis et al., 2011; Reniers et al., 2014), whereas rAG is associated with understanding others’ states (Schurz et al., 2017; Filmer et al., 2019; Lu et al., 2021). These mental processes play a pivotal role in lying or deception (Walczyk and Cockrell, 2022). Thus, stronger coupling between the rFPC and rAG may indicate that individuals try to know what others are thinking, which may contribute to generating an original lie.

The results of the playing tricks task were partially consistent with H2c: the NC increment of the rDLPFC–lIFG was lower during the playing tricks task than during the other tasks. Playing tricks may be associated with dark humor, which is characterized by transgression from social norms and moral systems (e.g., hurting or upsetting or demonstrating superiority over others; Aillaud and Piolat, 2012; Perchtold-Stefan et al., 2020). In addition, dark humor is positively correlated with MC (Perchtold‐Stefan et al., 2020). The rDLPFC is related to harnessing self-interest motivation (Knoch et al., 2006), whereas the lIFG is associated with cognitive humor processing (Samson et al., 2008; Bekinschtein et al., 2011; Vrticka et al., 2013). Thus, the lIFG may participate in the construction of fun tricks. Meanwhile, individuals may need to be more “selfish” to obtain fun. Therefore, the decrease in the NC increments of the rDLPFC–lIFG indicates that individuals focus on obtaining self-interested pleasures when generating a “dark” trick to upset others. Additionally, activation of the rDLPFC negatively predicts the harmfulness of playing tricks. In line with previous findings, this result suggests that controlling selfish motivation causes low harmfulness in the playing tricks task (Knoch et al., 2006). The results also showed that higher coupling between the right superior temporal gyrus and the rSMG significantly negatively predicted the originality of the playing tricks task. The right superior temporal gyrus is activated during humor processing (Shibata et al., 2014). Increased activity of the rSMG is linked to reduced emotional egocentricity (Silani et al., 2013). Thus, awareness of a victim’s emotions may hinder the construction of a novel trick. Thus, stronger coupling between the right superior temporal gyrus and rSMG may indicate lower originality of the playing tricks task.

The NC increment of the rDLPFC–rIPL was lower than that of the other MC tasks. The rDLPFC is also involved in controlling selfish motivation (Knoch et al., 2006). A previous study showed that the rIPL participates in maintaining the self-other distinction (Uddin et al., 2006). Individuals experience more distress induced by others if the self–other distinction is inadequate (Krol and Bartz, 2022). Considering that the hurting people task requires participants to hurt the victims directly (e.g., physical damage), participants may be more likely to realize the victims’ negative feelings. Thus, this NC result implies that individuals may need to release selfish motivation and avoid being affected by victims’ emotions during the hurting people task, which further maximizes the performance of hurting people. The involvement of the right precuneus, AG, and superior temporal gyrus (proposed in H2a) in the hurting people task was not different from that in other tasks, which might be because of the uniqueness of suppressing victims’ emotions when hurting people. Moreover, the performance of the hurting people task was negatively predicted by the activation of the rPOG and neural coupling of the lDLPFC–rPOG. Previous studies demonstrated that the rPOG is associated with empathy (Seehausen et al., 2016) and emotional perception (Gao et al., 2022a). The prefrontal cortex (including lDLPFC) plays a central role in appropriate moral behavior (Forbes and Grafman, 2010; Dashtestani et al., 2018). Therefore, low coupling between the lDLPFC and the rPOG indicates that individuals try to suppress the process of knowing the victim’s state and behave immorally, leading to more original and harmful ideas.

The results of linear regression analysis showed that neural activity in the three kinds of MC idea generation significantly predicted the level of Dark Triad, moral identity, and general and MC potential. Activation of the rIFG during the hurting people task negatively predicted the level of Dark Triad. Studies showed that the rIFG is involved in inhibition (Aron et al., 2003). Individuals with less inhibition toward immoral ideas during the hurting people task tended to have higher Dark Triad. The results revealed that moral identity was negatively predicted by the activity of the right fusiform gyrus during the hurting people and lying tasks. These results imply that individuals who actively reorient their attention toward malevolent task requirements may have low moral identity (Caspers et al., 2014). However, this effect was not significant in the playing tricks task, maybe because playing tricks was considered less morally related. Moreover, the results showed that activation and neural coupling within the prefrontal cortex were negatively related to general creativity potential in the three MC tasks. The activity of the prefrontal cortex is related to appropriate moral behavior (Forbes and Grafman, 2010; Dashtestani et al., 2018), which is contrary to the requirements of the three MC tasks. Nonobedience of creative task requirements may indicate less general creativity potential. As for the hurting people and playing tricks tasks, the neural coupling of rDLFPC–rPOG and rMOG–rSMG negatively predicted hurting people and playing tricks potential. The rDLPFC and rMOG are associated with prosocial mental processes, such as controlling selfish motivation (Knoch et al., 2006) and moral judgment (Qiao et al., 2022). The activities of the rPOG and rSMG are involved in the emotional perception of victims (Silani et al., 2013; Gao et al., 2023). Thus, the connectivity between these regions may weaken the generation of malevolent ideas. Another study found that neural coupling between the lDLPFC and rFPC positively predicted the lying potential. Previous studies showed that the rFPC is linked to well rehearsed lies (Ganis et al., 2003; Abe, 2011), whereas the ECN (including the lDLPFC) is important in idea generation (Beaty et al., 2016; Zhu et al., 2017). Consequently, stronger neural coupling between the lDLPFC and rFPC could be an indicator of a higher lying potential.

In general, these findings contribute to better understanding of the neural substrates underlying the generation of different creative malevolent ideas, which highlights the crucial role of the rFPC, rDLPFC, rIPL, and lIFG. Based on these findings, specific precautions should be taken for the different kinds of MC. For example, empathy interventions may be effective in preventing individuals from finding creative ways to hurt others. Moreover, the neural substrates of malevolent idea generation by criminals and terrorists should be further explored in the future. This may be helpful in predicting and preventing extremely harmful malevolent behaviors.

Several limitations should be mentioned, as follows. (1) fNIRS could only detect the outer cortex in the PFC and the right temporal and parietal regions. However, subcortical areas may also be involved in idea generation in different kinds of MC. Therefore, devices with higher spatial resolution (fMRI and MEG) should be used to explore the neural correlates of idea generation in different kinds of MC. (2) There was gender imbalance in this study sample. The effects of potential gender differences should be examined in the future. (3) This study focused only on MC in laboratory experiments. Whether the neural correlates of generating different malevolent creative ideas are dependent on the context (laboratory vs real life) should be further examined. (4) It should be noted that participants completed postexperimental scales after three MC tasks. Engagement in malevolent idea generation may influence participants’ subsequent evaluation of their behavior in daily life, which may affect the scores of scales. (5) Only limited variables (i.e., the Dark Triad of personality, moral personality, general and malevolent creativity potential) were measured in this study; other related variables (e.g., antagonism, state and trait anger, aggression, and emotion regulation) should be investigated in future studies to explore the relationship between these related variables and the neural substrates of different kinds of MC idea generation. Therefore, caution should be exercised when generalizing the findings of this study.

## Synthesis

Reviewing Editor: Ifat Levy, Yale School of Medicine Decisions are customarily a result of the Reviewing Editor and the peer reviewers coming together and discussing their recommendations until a consensus is reached. When revisions are invited, a fact-based synthesis statement explaining their decision and outlining what is needed to prepare a revision will be listed below. The following reviewer(s) agreed to reveal their identity: NONE.

This paper uses fNIRS to examine neural correlates of engaging in different types of malevolent creativity (hurting people, lying, playing tricks). The authors report similar prefrontal and temporal involvement for all three types of malevolent creativity, but also find specific brain activation patterns that may facilitate the unique attributes of each type of harmful creativity.

Investigating brain correlates of different types of malevolent creativity is interesting and the study is straightforward; however, there are issues with the current version of the manuscript, which should be addressed.

- While it is clear that the typology for malevolent creativity (i.e., the three types) are based on the Malevolent Creativity Behavior Scale (MCBS), reviewers did not agree with several of the authors’ strong statements that “there are three kinds of MC behaviors in daily life”. This sounds like this is the only classification and there are no other possibilities. However, the MCBS scheme of hurting, lying, and playing tricks is only one attempt to classify malevolent creativity behavior, and there may be several other ways to do so. The authors should tone down their statements on this classification and simply say that this is one possibility to categorize/classify malevolent creativity behavior.

- The literature is not up to date with respect to neuronal correlates of malevolent creative ideation. In early 2023, two EEG papers were published that describe EEG alpha activity correlates and functional coupling during malevolent creative ideation and provide in-depth reflections on the meaning of this cortical activations regarding harmful ideation:

Perchtold-Stefan, C. M., Rominger, C., Papousek, I., & Fink, A. (2023). Functional EEG Alpha Activation Patterns During Malevolent Creativity. Neuroscience, 522, 98-108.

Perchtold-Stefan, C. M., Rominger, C., Papousek, I., & Fink, A. (2023). Women and men have a similar potential for malevolent creativity-But their underlying brain mechanisms are different. Brain Research, 1801, 148201.

These studies should be incorporated into the literature background and considered for the interpretation of results, as they may be very helpful to properly interpret this study’s findings.

- Throughout the manuscript, the authors consistently mention malevolent creativity in relationship to criminal activity or terrorist activity. This suggests that this study will test a criminal/clinical sample, however ultimately, only a non-clinical student sample is tested. The authors are advised to focus the introduction more on explaining why malevolent creativity may also be highly relevant in the general population and how “smaller scale” malevolent creativity behaviors may negatively impact society without necessarily going into criminal territory.

- Did the authors apply some difficulty ratings (as in, participants rated how difficult each of the tasks was for them)? It seems that generating creative ideas for playing tricks would be more difficult than generating creative ways of hurting others, for example. How do we know that these tasks are comparable with regard to cognitive effort?

- Post-experimental tasks: There are two issues there.

First, it is unclear why the authors choose to implement these exact post-experimental measures, like the Honesty- Humility Inventory or the Dirty Dozen? What are the exact expectations here? Why these measures and not other measures shown to be relevant in the literature (like antagonism, state and trait anger, aggression, morality, emotion regulation, etc.?). The choice of questionnaires needs to be explained regarding their relevance to the present study.

Second, it may be problematic that self-report measures on general and malevolent creativity behavior in daily life were administered after the malevolent creativity fNIRS tasks, and not before. This way, prolonged engagement in the malevolent creativity tasks may have influenced participants’ estimation of their creative behavior in daily life. Thus, the questionnaires scores are likely not fully reflective of their natural creative behavior. This is a limitation that, at the minimum, should be elaborated on in the discussion section.

- Statistical analysis: ANCOVA (for each task and tasks comparisons) and linear regression analysis may be better than separate tests for showing the relation between beta increments, task indices (pre and post) and neural coupling.

- An overview summary figure, summarizing and integrating the findings will be helpful.

- Discussion, page 22 to 23, lines 464 zo 465: “Although playing tricks is less harmful than other MC, it selfishly gets others into bad situations. A good trick must consider victim’s emotions”. Please elaborate on this interpretation, as it is not clear what you mean.

- Discussion: It should be discussed why no correlations between beta increments and post-experimental measures were observed, like the dirty dozen or honesty-humility, and importantly, scores on the malevolent creativity behavior scale. Isn’t it expected that personality traits like psychopathy or low honesty and self-reported MC behavior will influence brain activation during malevolent creative ideation? Since this is not the case, it raises the question whether the three tasks really resemble malevolent creativity in daily life, or whether, given the lack of correlations with personality, it is a rather hypothetical, less realistic approach to malevolent creativity? The authors should discuss this. In particular, the lack of correlation between the MCBS and brain activation during malevolent creativity tasks based on the MCBS is concerning.

- More generally, the discussion of limitations and future directions should be extended.

## Author Response

Response to Reviews

Dear Dr. Ifat Levy and reviewers,

We really appreciate your patient work and profound advice on our manuscript “Similarities and distinctions between cortical neural substrates that underlie generation of malevolent creative ideas (eN-NWR-0127-23)”, and we are quite grateful that you have decided to give us the precious opportunity to resubmit. To further improve the readability, the manuscript was polished by native English speaker. Here is our response to the editor and reviewers.

Comment 1

- Please remove “University Committee on Human Research Protection of the East

143 China Normal University (HR 039-2017)” from page 8 of the manuscript.

In order to facilitate the double-blind review process, no information may be present that might reveal the identity of any author. If you feel this information is essential to the review of the manuscript, then please reply to this email to inquire whether your manuscript may proceed with a non-double-blind review process.

Response：

Thanks for your reminding! We have covered the information that might reveal our identity. See details in 2.1.1. Participants, Page 7-8.

Revision:

In 2.1.1. Participants, Page 7-8:

The study procedure was approved by the University Committee on Human Research Protection of the ******** (Code ********).

Comment 2

Computational Neuroscience Model Code Accessibility Comments for Author (Required):

Please include a statement in the Materials and Methods section, under the heading “Code Accessibility”, indicating whether and how analysis codes can be accessed.

Response:

Thanks for your reminding! We have added the “Code Accessibility” into Methods, see details in 2.10. Code Accessibility, Page 15.

Revision:

See details in 2.10. Code accessibility, Page 15:

The code used in this study is available from the corresponding author upon request.

Comment 3

Synthesis Statement for Author (Required):

This paper uses fNIRS to examine neural correlates of engaging in different types of malevolent creativity (hurting people, lying, playing tricks). The authors report similar prefrontal and temporal involvement for all three types of malevolent creativity, but also find specific brain activation patterns that may facilitate the unique attributes of each type of harmful creativity.

Investigating brain correlates of different types of malevolent creativity is interesting and the study is straightforward; however, there are issues with the current version of the manuscript, which should be addressed.

- While it is clear that the typology for malevolent creativity (i.e., the three types) are based on the Malevolent Creativity Behavior Scale (MCBS), reviewers did not agree with several of the authors’ strong statements that “there are three kinds of MC behaviors in daily life”. This sounds like this is the only classification and there are no other possibilities. However, the MCBS scheme of hurting, lying, and playing tricks is only one attempt to classify malevolent creativity behavior, and there may be several other ways to do so. The authors should tone down their statements on this classification and simply say that this is one possibility to categorize/classify malevolent creativity behavior.

Response:

Thanks for your delightful suggestion! We have revised our statement to explicitly declared that the classification based on MCBS was only one possible classification of MC behaviors. See details in Introduction, Page 4.

Revision:

In Introduction, Page 4:

However, MC is not a one-dimensional framework. Researchers have proposed a possible classification of MC behaviors in daily life, namely hurting people, lying, and playing tricks (Hao et al., 2016).

Comment 4

- The literature is not up to date with respect to neuronal correlates of malevolent creative ideation. In early 2023, two EEG papers were published that describe EEG alpha activity correlates and functional coupling during malevolent creative ideation and provide in-depth reflections on the meaning of this cortical activations regarding harmful ideation:

Perchtold-Stefan, C. M., Rominger, C., Papousek, I., & Fink, A. (2023). Functional EEG Alpha Activation Patterns During Malevolent Creativity. Neuroscience, 522, 98-108.

Perchtold-Stefan, C. M., Rominger, C., Papousek, I., & Fink, A. (2023). Women and men have a similar potential for malevolent creativity-But their underlying brain mechanisms are different. Brain Research, 1801, 148201.

These studies should be incorporated into the literature background and considered for the interpretation of results, as they may be very helpful to properly interpret this study’s findings.

Response:

Thanks for your delightful suggestion! We have added these two recent studies to enrich our manuscript. See details in Introduction, Pages 3-4.

Revision:

For example, see details in Introduction, Pages 3-4:

Perchtold-Stefan et al. (2023a) used an EEG device to investigate task-related power changes in the alpha band during the generation of malevolent ideas. The results showed that individuals with higher MC performance had increased prefrontal alpha power in the early stage (indicating conceptual expansion from prosocial to antisocial) and increased temporal alpha power in the middle stage (indicating inhibition of dominant but normal ideas; Perchtold-Stefan et al., 2023a). Furthermore, researchers have noticed gender differences in the neural substrates underlying MC. Although females and males have similar levels of MC performance, females tend to generate malevolent creative ideas through semantic memory retrieval and the recombination of social information, whereas males rely more on motor-related imagery (Perchtold-Stefan et al., 2023b).

Comment 5

- Throughout the manuscript, the authors consistently mention malevolent creativity in relationship to criminal activity or terrorist activity. This suggests that this study will test a criminal/clinical sample, however ultimately, only a non-clinical student sample is tested. The authors are advised to focus the introduction more on explaining why malevolent creativity may also be highly relevant in the general population and how “smaller scale” malevolent creativity behaviors may negatively impact society without necessarily going into criminal territory.

Response:

Thanks for your kind and delightful suggestion! We reduced the focus on the extreme population (i.e., terrorists and criminals) and emphasized more on the general population (i.e., non-clinical/criminal sample) in the whole manuscript. Moreover, more statements were added in Introduction to explain the meaning of exploring malevolent creativity in general population.

We really agree that investigating the malevolent creativity in extreme population is intriguing and important. Thus, we will advise researcher to further explore the malevolent creation in extreme population in the future. See details in Discussion.

Revision

For example, in Introduction, Page 2:

Although MC is usually marked by creative criminal or terrorism activities (e.g., Cropley & Cropley et al., 2008; Gill et al., 2013), it can be observed in the general population (e.g., Hao et al., 2020; Perchtold-Stefan et al., 2020a), such as framing other colleagues creatively to avoid punishment. These MC behaviors may not be considered crimes but still hurt others’ personal interests. Most people are more likely to encounter the outcomes of MC from ordinary people than from criminals or terrorists. Additionally, non-criminal malevolent creative behavior may escalate into criminal malevolent creative behavior in the future. Therefore, based on the findings from studies on criminals or terrorism, researchers have tried to understand MC in the general population.

In Discussion, Page 25:

Moreover, the neural substrates of malevolent idea generation by criminals and terrorists should be further explored in the future. This may be helpful in predicting and preventing extremely harmful malevolent behaviors.

Comment 6

- Did the authors apply some difficulty ratings (as in, participants rated how difficult each of the tasks was for them)? It seems that generating creative ideas for playing tricks would be more difficult than generating creative ways of hurting others, for example. How do we know that these tasks are comparable with regard to cognitive effort?

Response:

Thanks for your kind reminding! These three kinds of malevolent tasks were adopted from the previous study. It showed that there were no significant differences in the aspects of difficulty, fatigue, involvement and familiarity among these three MC tasks (Cheng et al., 2021b). We added the details in the main text.

Revision:

In 2.3. Experimental tasks, Page 8-9:

The hurting people tasks, lying tasks and playing tricks tasks were adopted from the previous study. It showed that there were no significant differences in the aspects of difficulty, fatigue, involvement and familiarity among these three MC tasks (Cheng et al., 2021b).

Cheng, R., Lu, K., & Hao, N. (2021b). The effects of anger on different forms of malevolent creative performance. Journal of Psychological Science, 44, 1336-1345.

Comment 7

- Post-experimental tasks: There are two issues there.

First, it is unclear why the authors choose to implement these exact post-experimental measures, like the Honesty- Humility Inventory or the Dirty Dozen? What are the exact expectations here? Why these measures and not other measures shown to be relevant in the literature (like antagonism, state and trait anger, aggression, morality, emotion regulation, etc.?). The choice of questionnaires needs to be explained regarding their relevance to the present study.

Response:

Thanks for your suggestion! We have cited more studies and added more statement in Introduction (Page 7) to explain why we choice these post-experimental measures. We agree with you that there are several other variables (e.g., trait anger) may relevant to malevolent creativity. In Limitation (Page 26), we have reminded the reader to noticed that there were other variables that related to MC, as well as advised researcher to further explore the relation between other related variables and neural substrates of malevolent creativity.

Revision:

In Introduction, Page 7:

Moreover, previous studies found that the Dark Triad of personality, moral personality, general creativity potential, and MC potential can affect MC performance (Gao et al., 2022b; Jonason et al., 2017; Kapoor & Kaufman, 2022b; Perchtold-Stefan et al., 2023). Therefore, we also measured these indices using a series of scales and tested whether these variables were distinctively related to the neural substrates of the different kinds of MC idea generation.

In Limitation Page 26:

... (5) Only limited variables (i.e., the Dark Triad of personality, moral personality, general and malevolent creativity potential) were measured in this study; other related variables (e.g., antagonism, state and trait anger, aggression, and emotion regulation) should be investigated in future studies to explore the relationship between these related variables and the neural substrates of different kinds of MC idea generation.

Comment 8

Second, it may be problematic that self-report measures on general and malevolent creativity behavior in daily life were administered after the malevolent creativity fNIRS tasks, and not before. This way, prolonged engagement in the malevolent creativity tasks may have influenced participants’ estimation of their creative behavior in daily life. Thus, the questionnaires scores are likely not fully reflective of their natural creative behavior. This is a limitation that, at the minimum, should be elaborated on in the discussion section.

Response:

Thanks for your reminding! We added the relevant contents into Discussion to reminded the readers about this limitation. See details in Discussion, Page 26.

Revision:

In Discussion, Page 26

... (4) It should be noted that participants completed post-experimental scales after three MC tasks. Engagement in malevolent idea generation may influence participants’ subsequent evaluation of their behavior in daily life, which may affect the scores of scales.

Comment 9

- Statistical analysis: ANCOVA (for each task and tasks comparisons) and linear regression analysis may be better than separate tests for showing the relation between beta increments, task indices (pre and post) and neural coupling.

Response：

Thanks for your helpful and delightful suggestion! First, we entered all the predictors (i.e., neural activations of all channel or neural couplings between all channel combinations) into the linear regression model, but the coefficients of predictors could not be calculated due to the problem of rank deficiency (i.e., the quantity of predictors strongly outnumber sample size). Therefore, we used several Pearson correlation analyses to primarily selected the channels (neural activation) or channel combinations (neural coupling) that significantly correlated with task performances (i.e., the originality and harmfulness of hurting people, lying and playing trick tasks). Then, linear regression analysis was conducted on the selected channels or channel combinations to captured the significant results. See details in Method (2.8. Data analysis of the neural activation; 2.9. Data analysis of neural coupling) and Results.

For task comparisons of beta increment and neural coupling, we first used false discovery rate correction method (FDR; corrected alpha level = 0.05) to avoid the false positive results. Next, the anxiety and pleasure of three tasks were considered as covariates because the level of anxiety and pleasure were significantly different across the three tasks. Given that pleasure and anxiety only related to the corresponding malevolent creativity task (e.g., pleasure of lying tasks related to lying task instead of hurting people or playing trick tasks), we have introduced linear mix model to control the potential effect of covariates on the significant results. After controlling the effects of covariates, the results still remained significant. We believed that the abovementioned procedures were efficient to control the potential effects from covariates.

Revision:

For example:

In 2.8. Data analysis of the neural activation, Page 14:

Linear regression was performed to quantify the relationship between beta increments from all the channels and task performance. Before the formal linear regression procedure, several Pearson correlation analyses were performed on the behavioral performance and beta increments of each channel. Only the channels that were significantly correlated with behavioral performance (p ＜. 05, uncorrected) were introduced into the subsequent analysis as predictors (Xie et al., 2022). In this study, linear regression was performed on every behavioral performance (with beta increments as predictors). In addition, the relationships between the post-experimental scales and beta increments were examined using linear regression (with post-experimental scales as dependent variables and beta increments as predictors).

In 3.4.1. The originality and harmfulness of MC tasks, Page 19 Linear regression analysis was used to quantify the relationship between neural results (beta increments and neural couplings) under different conditions and task performances (i.e., originality and harmfulness of the hurting people, lying, and playing tricks tasks). The results revealed that for the hurting people task, harmfulness was negatively predicted by the activation of rPOG (CH30; b = -19.70, 95%CI = [.15, 35.58], F = 6.03, p = .005) and originality was negatively predicted by the neural coupling of lDLPFC-rPOG (CH5-CH35; b = -1.65, 95%CI = [-2.78, -.53], F = 10.09, p ＜ .001). For the lying task, originality was positively predicted by the neural coupling between rFPC and rAG (CH12-CH35; b = 1.60, 95%CI = [.47, 2.73], F = 5.50, p ＜ .001). For the playing tricks task, harmfulness was negatively predicted by the activation of rDLPFC (CH21; b = -20.94, 95%CI = [-40.85, -1.03], F = 4.53, p = .040) and originality was negatively predicted by the neural coupling between the right superior temporal gyrus and rSMG (CH26-34; b = -.80, 95%CI = [-1.51, -.08], F = 6.78, p ＜ .001).

Comment 10

- An overview summary figure, summarizing and integrating the findings will be helpful.

Response:

Thanks for your reminding! We have added the summary figures. See details in Figures 4A-4D.

Revision:

Figure 4

Comment 11

- Discussion, page 22 to 23, lines 464 zo 465: “Although playing tricks is less harmful than other MC, it selfishly gets others into bad situations. A good trick must consider victim’s emotions”. Please elaborate on this interpretation, as it is not clear what you mean.

Response:

Thanks for your helpful suggestion! Given that we have re-examined the relation between neural activity (i.e., neural activation and coupling) by linear regression analysis (see details in Comment 9), the original sentence was removed. See details in Discussion.

Comment 12

- Discussion: It should be discussed why no correlations between beta increments and post-experimental measures were observed, like the dirty dozen or honesty-humility, and importantly, scores on the malevolent creativity behavior scale. Isn’t it expected that personality traits like psychopathy or low honesty and self-reported MC behavior will influence brain activation during malevolent creative ideation? Since this is not the case, it raises the question whether the three tasks really resemble malevolent creativity in daily life, or whether, given the lack of correlations with personality, it is a rather hypothetical, less realistic approach to malevolent creativity? The authors should discuss this. In particular, the lack of correlation between the MCBS and brain activation during malevolent creativity tasks based on the MCBS is concerning.

Response:

Thanks for your useful and delightful reminding! Based on Comment 9, we used linear regression instead of separate Pearson correlation to re-analyze the relation between neural activity (i.e., neural activation and coupling) and post-experimental scales. We have found some significant results and explained its meaning, see details in Method (2.8. Data analysis of the neural activation, Page 13-14; 2.9. Data analysis of neural coupling, Page 14-15), Results (3.4.2. Post-experimental scales, Page 19-20) and Discussion.

Revision:

For example, 2.8. Data analysis of the neural activation, Page 14

... In addition, the relationships between the post-experimental scales and beta increments were examined using linear regression (with post-experimental scales as dependent variables and beta increments as predictors).

Comment 13

- More generally, the discussion of limitations and future directions should be extended.

Response:

Thanks for your kind reminding! We have extended the contents about limitation and future study directions in Discussion.

Eventually, we really appreciate your patient work and profound advice on our manuscript. We think that the manuscript is now much improved and we are really willing to make any further revisions. It would be a great honor that our paper could be accepted by eNeuro. Thank you!

Your sincerely,

Ning Hao
